# Risk of cardiovascular disease in patients with alcohol use disorder: A population-based retrospective cohort study

**DOI:** 10.1371/journal.pone.0276690

**Published:** 2022-10-25

**Authors:** Chieh Sung, Chi-Hsiang Chung, Fu-Huang Lin, Wu-Chien Chien, Chien-An Sun, Chang-Huei Tsao, Chih-Erh Weng

**Affiliations:** 1 Graduate Institute of Medical Sciences, National Defense Medical Center, Taipei, Taiwan; 2 Department of Nursing, Keelung Chang Gung Memorial Hospital, Keelung, Taiwan; 3 School of Public Health, National Defense Medical Center, Taipei, Taiwan; 4 Taiwanese Injury Prevention and Safety Promotion Association, Taipei, Taiwan; 5 Graduate Institute of Life Sciences, National Defense Medical Center, Taipei, Taiwan; 6 Department of Medical Research, Tri-Service General Hospital, National Defense Medical Center, Taipei, Taiwan; 7 Department of Public Health, College of Medicine, Fu-Jen Catholic University, New Taipei City, Taiwan; 8 Big Data Research Center, College of Medicine, Fu-Jen Catholic University, New Taipei City, Taiwan; 9 Department of Medical Research, Tri-Service General Hospital, Taipei City, Taiwan; 10 Department of Microbiology & Immunology, National Defense Medical Center, Taipei City, Taiwan; Tungs’ Taichung MetroHarbor Hospital, TAIWAN

## Abstract

The complex effects of alcohol consumption on the cardiovascular system vary with mean daily consumption and duration of intake. This population-based retrospective cohort study aimed to explore the risk of cardiovascular disease (CVD) in patients with alcohol use disorder (AUD). Data was collected from the Taiwan National Health Insurance Research Database from 2000 to 2013. A total of 7,420 patients with AUD were included in our study group, and 29,680 age- and sex-matched controls without AUD in the control group. Cox proportional hazard regression analysis was used to investigate the effects of AUD on the risk of CVD. Most patients were men aged 25–44 years. At the end of the follow-up period, the AUD group had a significantly higher incidence of CVD (27.39% vs. 19.97%, P<0.001) and more comorbidities than the control group. The AUD group also exhibited a significantly higher incidence of CVD than the control group based on the Cox regression analysis and Fine and Gray’s competing risk model (adjusted hazard ratio [AHR] = 1.447, 95% confidence interval [CI] = 1.372–1.52 5, P<0.001). Furthermore, male sex, diabetes mellitus, hypertension, hyperlipidemia, chronic kidney disease, chronic obstructive pulmonary disease, anxiety, depression, and a high Charlson Comorbidity Index were also associated with an increased risk of CVD. Patients with AUD in different CVD subgroups, such as those with CVD, ischemic heart disease (IHD), and stroke, were at a significantly higher risk of disease than those without AUD; CVD (AHR = 1.447, 95% CI = 1.372–1.525, P<0.001), IHD (AHR = 1.304, 95% CI = 1.214–1.401, P<0.001), and stroke (AHR = 1.640, 95% CI = 1.519–1.770, P<0.001). The risk also significantly differed among patients in the different CVD subgroups. We observed an association between AUD and development of CVD even after adjusting for several comorbidities and medications in our nationwide population cohort.

## Introduction

Alcohol use disorder (AUD) is characterized by compulsive alcohol seeking, loss of control with regard to limiting intake, and persistent alcohol use despite awareness of the harmful consequences such as alcoholic liver disease, cancer, cardiovascular disease (CVD), cirrhosis, and neuropsychiatric disorders [1–3].

Heterogeneous associations exist between the level of alcohol consumption and the initial presentation of CVD. Previous studies indicate that low-to-moderate levels of alcohol consumption could reduce the risk of most CVDs. Thus, the relationship between alcohol consumption and CVDs is complex and controversial [4]. Hence, to enhance the understanding of the risk of CVDs associated with AUD, we conducted a large, nationwide, population-based nested cohort study using Taiwan’s National Health Insurance Research Database (NHIRD).

## Methods

### Data source

The National Health Insurance Program (NHI) was launched in Taiwan in 1995 and covers more than 99% of the Taiwanese population (more than 23 million beneficiaries). The NHIRD contains the following encrypted data: patient identification number; date of birth; sex; dates of admission and discharge; worldwide class of sicknesses, 9th Revision, medical modification (ICD-nine-CM) diagnostic and system codes (as many as 5 each); and outcomes. The longitudinal medical health insurance Database 2005 (LHID 2005), which we used, is a subset of the NHIRD. The LHID 2005 carries approximately 1 million randomly selected records of beneficiaries, representing approximately 5% of the population in Taiwan in 2005, for scientific utilization. Statistics from 2000–2013 were extracted from the NHIRD.

Analysis of data from 2000 to 2013 using the Universal Health Coverage database, Tandem「Inpatient expenditures by admissions (DD)」、「Registry for contracted medical facilities (HOSB) 」、「Registry for beneficiaries(ID)」 、「Registry for catastrophic illness patients(HV) 」, variables include diagnosis, surgery, disposition, hospitalization and discharge dates, length of stay and medical costs; 「Registry for contracted medical facilities (HOSB) 」the variables include hospital location and hospital level.

According to the law, medical institutions are required to report outpatient (including emergency) and inpatient expenses to the Health Insurance Bureau every month. Therefore, Therefore, health insurance data is a representative empirical data in the field of medical and health-related research, and analytical results thereof can be used as a reference for medical and health policies, providing an important research resource. The NHI Administration periodically reviews medical records in a random manner to verify the accuracy of diagnoses. This review was conducted in accordance with the World Medical Association Code of Ethics (Helsinki Declaration). This study was approved by the Institutional Review Board of Tri-Service General Hospital at the National Defense Medical Center in Taipei, Taiwan, and the requirement of individual consent was waived because all identifying data were encrypted (TSGH IRB No. B-111-10). The NHIRD is a publicly available database that contains depersonalized patient information to ensure patient anonymity.

### Study sample

**Fig 1:**
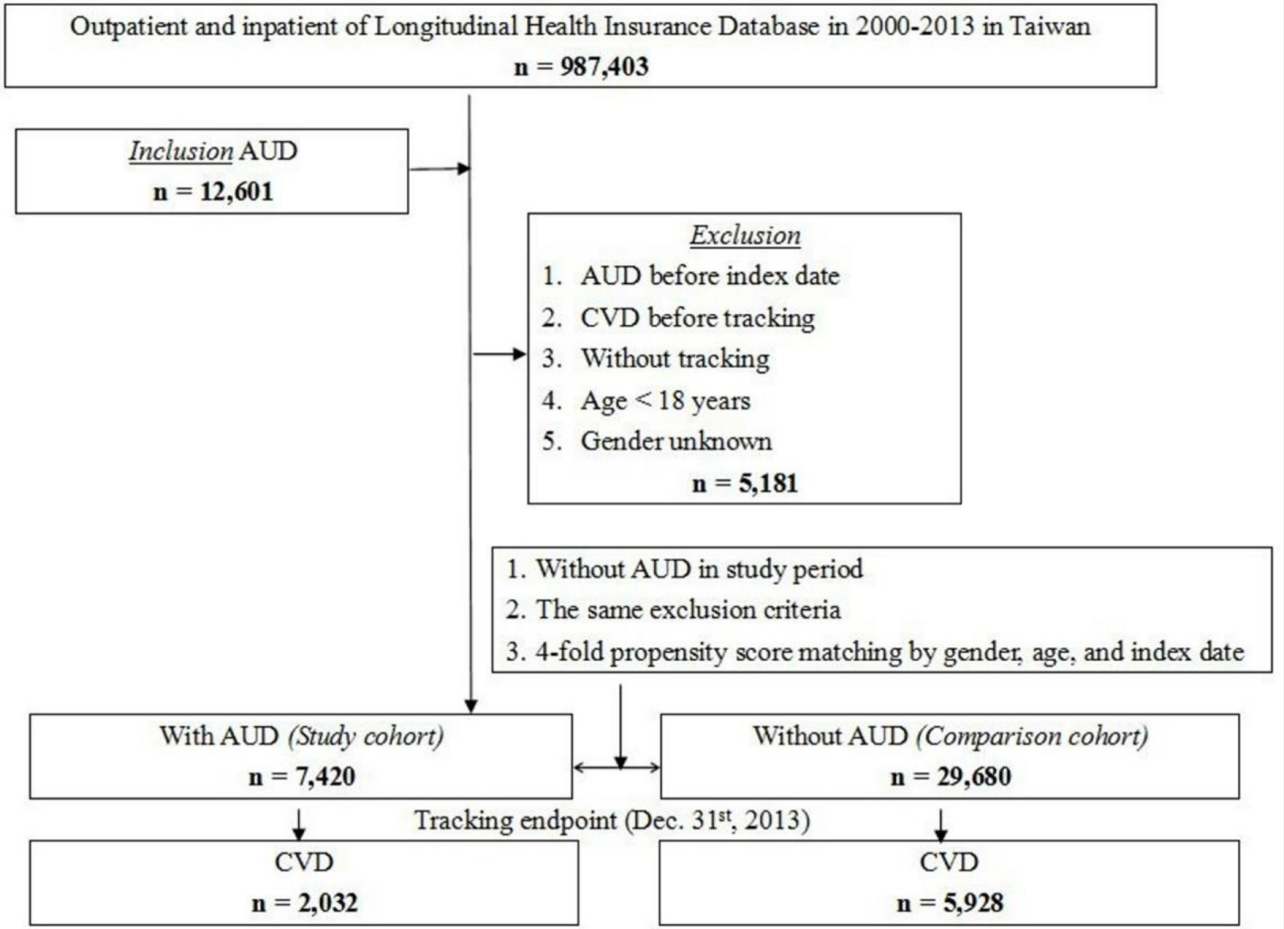
The flowchart of study sample selection from the National Health Insurance Research Database in Taiwan. Abbreviations: AUD, alochol use disorder; CVD, cardiovascular disease.

The study comprised a cohort of patients aged above 18 years from the LHID 2005 database who were newly diagnosed with alcohol use disorder, namely alcoholic psychosis (ICD-9-CM 291), alcohol abuse (ICD-9-CM 303.0, 305.0), and alcohol dependency syndrome (ICD-9-CM 303.9). We utilized the LHID to estimate the incidence of alcohol-related illnesses as previously specified in the Centers for Disease Control and Prevention’s "Chronic Causes" of "Alcohol-Related ICD Codes"(https://nccd.cdc.gov/DPH/ARDI/Info/ICDCodes.aspx) and as previously documented in the literature [5,6]. CVD was identified using the codes for ischemic heart disease (IHD) (ICD-9-CM 410–414) and stroke (ICD-9-CM 430–438). We excluded patients with a history of AUD, aged <18 years of age, whose sex was unknown, who had CVD before tracking, with incomplete tracking data, and who were diagnosed with AUD before the index date, the inclusion and exclusion criteria are shown in Fig 1. Those who comprised the control group were also selected from the LHID 2005. The study and control cohorts were selected with 1:4 matching according to sex, age, and index date. The date of the diagnosis of an alcohol-related disease was used as the index date.

### Outcome measurement and comorbidities

Patients with baseline comorbidities, IHD (ICD-9-CM 410–414), stroke (ICD-9-CM 430–438), diabetes mellitus (DM) (ICD-9-CM 250), hyperlipidemia (ICD-9-CM 272.0–272.4), hypertension (HTN) (ICD-9-CM 401–405), obesity (ICD-9-CM 278), depression (ICD-9-CM296.2e296.3, 300.4), anxiety (ICD-9-CM 300.02), chronic kidney disease (CKD) (ICD-9-CM 585), chronic obstructive pulmonary disease (COPD) (ICD-9-CM 490e496), liver cirrhosis (ICD-9-CM571), tobacco use disorder (ICD-9-CM350.1), and drug use disorder (ICD-9-CM304, 305.2–305.9), are listed in S1 Table.

All patients were followed up from the index date until the first diagnosis of CVD, death, withdrawal from the NHI program, or 31 December 2013. The covariates included sex, age group (18–24, 25–44, 45–64, ≥65), geographical area of residence (), urbanization level of residence (levels 1–4), low-income, catastrophic illness, Charlson comorbidity index—revised (CCI_R), season at diagnosis of CVD (spring, summer, autumn, winter), level of care (hospital center, regional hospital, local hospital). The urbanization level of residence was defined according to the population and various indicators of development: level 1 was defined as a population >1,250,000, with a specific designation of political, economic, cultural, and metropolitan development; level 2 was defined as a population of 500,000–1,249,999, with an important role in politics, economy, and culture; levels 3 and 4 were defined as populations of 149,999–499,999 and <149,999, respectively.

### Statistical analysis

The clinical characteristics of patients enrolled in the study are expressed in numerical form. We compared the distribution of categorical characteristics and baseline comorbidities between the case and control groups using Fisher’s exact test and the chi-squared test. Continuous variables are presented as means and standard deviations and were compared using t-tests. As the primary goal of the study was to determine whether the clinical characteristics of the patients were associated with the development of CVD, Fine and Gray’s survival analysis and regression analysis were used to determine the risk of CVD (competing with mortality), and the results are presented as hazard ratios (HRs) with the associated 95% confidence intervals (CIs). Associations between time-to-event outcomes and clinical characteristics were examined using the Kaplan–Meier method and multivariate Cox regression analysis with stepwise selection. The results are presented as adjusted HRs with the corresponding 95% CIs. All statistical analyses were performed using IBM SPSS Statistics for Windows version 22.0. (released 2013, IBM Corp., Armonk, NY, USA). A two-tailed P-value of <0.05 was considered statistically significant.

## Results

Among the 987,403 patients in the LHID 2000–2013, 12,601 were diagnosed with AUD; 7,420 patients were assigned to the study cohort and 29,680 age-, sex-, and comorbidity-matched patients were assigned to the comparison (control) cohort (Fig 1).

The baseline data of the patient and control groups are shown in Table 1. The patients were predominantly men (92.84%), with an average age of 43.12 ± 11.85 years. Our findings revealed that low-income, DM, liver cirrhosis, CKD, drug use disorder, anxiety, depression, location, Urbanization level, and level of care significantly differed between the study and control groups. In most patients with alcohol-related diseases, the diseases were diagnosed and treated in northern Taiwan, and middle Taiwan, with a combination of urbanization level 1 and 2 cities, and these patients were predominantly treated in regional hospital, or a local hospital. There were no significant differences in sex, age, CCI, and season between the groups.

**Table 1:** Characteristics of the patient and control groups at baseline.

AUD	Total		With		Without		P
Variables	n	%	n	%	n	%	
**Total**	37,100		7,420	20.00	29,680	80.00	
**Sex**							0.999
Male	34,445	92.84	6,889	92.84	27,556	92.84	
Female	2,655	7.16	531	7.16	2,124	7.16	
**Age (mean** ± **SD, y)**	43.29 ± 13.19		43.12 ± 11.85		43.32 ± 13.50		0.223
**Age groups (y)**							0.999
18–24	1,115	3.01	223	3.01	892	3.01	
25–44	22,075	59.50	4,415	59.50	17,660	59.50	
45–64	11,695	31.52	2,339	31.52	9,356	31.52	
≥65	2,215	5.97	443	5.97	1,772	5.97	
**Low-income**							<0.001
Without	36,675	98.85	7,300	98.38	29,375	98.97	
With	425	1.15	120	1.62	305	1.03	
**Catastrophic illness**							0.638
Without	34,040	91.75	6,798	91.62	27,242	91.79	
With	3,060	8.25	622	8.38	2,438	8.21	
**DM**							<0.001
Without	34,586	93.22	6,781	91.39	27,805	93.68	
With	2,514	6.78	639	8.61	1,875	6.32	
**HTN**							0.219
Without	35,078	94.55	6,994	94.26	28,084	94.62	
With	2,022	5.45	426	5.74	1,596	5.38	
**Hyperlipidemia**							<0.001
Without	36,107	97.32	7,066	95.23	29,041	97.85	
With	993	2.68	354	4.77	639	2.15	
**Obesity**							0.901
Without	37,084	99.96	7,417	99.96	29,667	99.96	
With	16	0.04	3	0.04	13	0.04	
**Liver cirrhosis**							<0.001
Without	32,111	86.55	4,467	60.20	27,644	93.14	
With	4,989	13.45	2,953	39.80	2,036	6.86	
**CKD**							<0.001
Without	36,224	97.64	7,294	98.30	28,930	97.47	
With	876	2.36	126	1.70	750	2.53	
**COPD**							0.756
Without	36,055	97.18	7,207	97.13	28,848	97.20	
With	1,045	2.82	213	2.87	832	2.80	
**Tobacco use disorder**							0.617
Without	37,099	100.00	7,420	100.00	29,679	100.00	
With	1	0.00	0	0.00	1	0.00	
**Drug use disorder**							<0.001
Without	37,021	99.79	7,358	99.16	29,663	99.94	
With	79	0.21	62	0.84	17	0.06	
**Anxiety**							<0.001
Without	36,961	99.63	7,359	99.18	29,602	99.74	
With	139	0.37	61	0.82	78	0.26	
**Depression**							<0.001
Without	36,590	98.63	6,980	94.07	29,610	99.76	
With	510	1.37	440	5.93	70	0.24	
**CCI_R**	0.40 ± 1.44		0.44 ± 1.09		0.39 ± 1.52		0.935
**Season**							0.999
Spring (Mar–May)	9,155	24.68	1,831	24.68	7,324	24.68	
Summer (Jun–Aug)	8,745	23.57	1,749	23.57	6,996	23.57	
Autumn (Sep–Nov)	9,320	25.12	1,864	25.12	7,456	25.12	
Winter (Dec–Feb)	9,880	26.63	1,976	26.63	7,904	26.63	
**Location**							<0.001
Northern Taiwan	14,715	39.66	2,823	38.05	11,892	40.07	
Middle Taiwan	10,284	27.72	2,149	28.96	8,135	27.41	
Southern Taiwan	9,422	25.40	1,683	22.68	7,739	26.07	
Eastern Taiwan	2,501	6.74	715	9.64	1,786	6.02	
Outlets islands	178	0.48	50	0.67	128	0.43	
**Urbanization level**							<0.001
1 (highest)	12,782	34.45	2,416	32.56	10,366	34.93	
2	14,876	40.10	2,933	39.53	11,943	40.24	
3	3,290	8.87	668	9.00	2,622	8.83	
4 (lowest)	6,152	16.58	1,403	18.91	4,749	16.00	
**Level of care**							<0.001
Hospital center	10,701	28.84	1,742	23.48	8,959	30.19	
Regional hospital	12,466	33.60	3,228	43.50	9,238	31.13	
Local hospital	13,933	37.56	2,450	33.02	11,483	38.69	

P: Chi-squared/Fisher’s exact test for categorical variables and t-test for continuous variables. AUD = Alcohol use disorder, DM = diabetes mellitus, HTN = hypertension, COPD = chronic obstructive pulmonary disease, CKD = chronic kidney disease, CCI = Charlson comorbidity index, SD = standard deviation.

**Fig 2:**
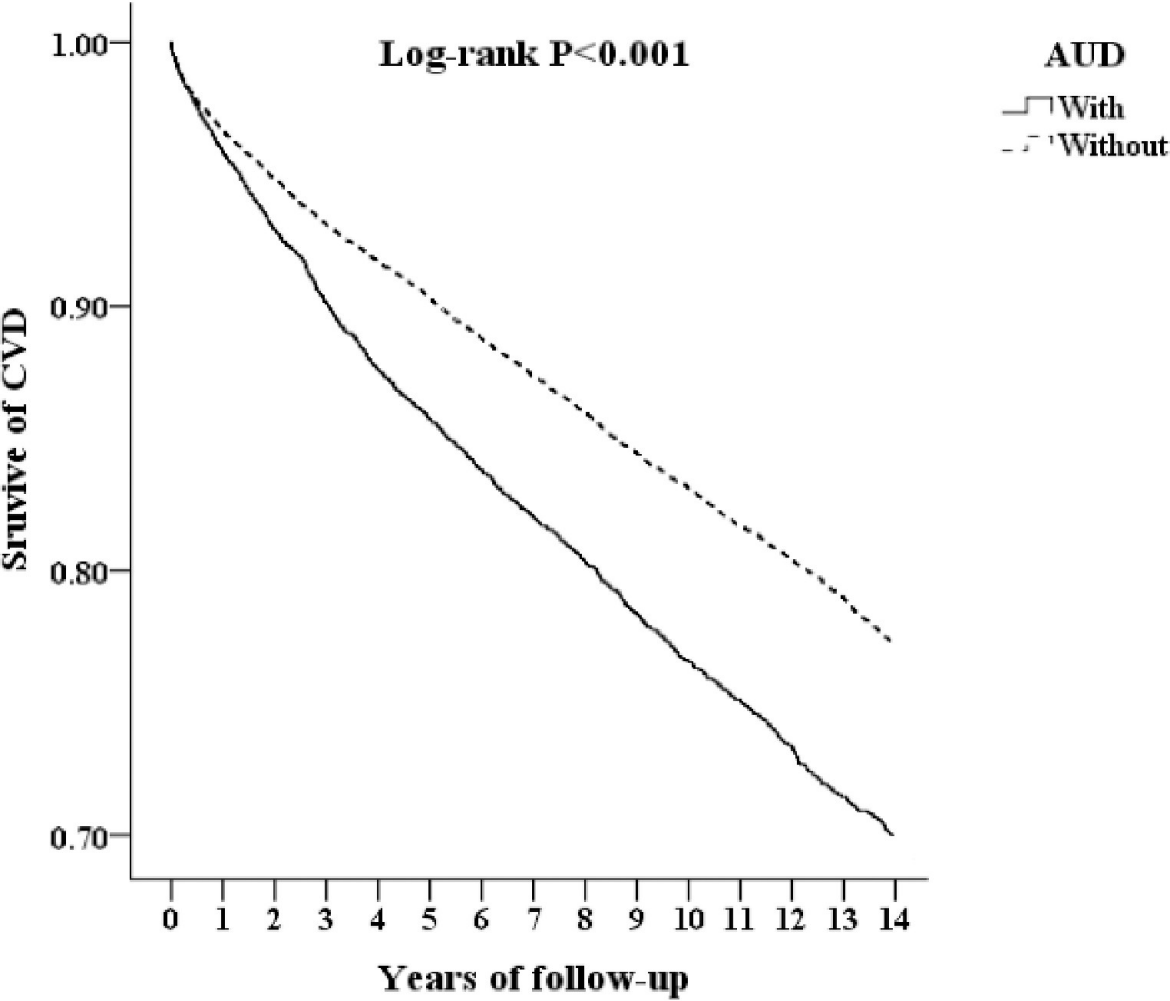
Kaplan–Meier curve of the CVD due to alcohol-related diseases. Abbreviations: CVD, cardiovascular disease; AUD, alchol use disorder.

Fig 2 shows the Kaplan–Meier survival curve of patients with CVD stratified by AUD using the log-rank test; patients with AUD had a significantly higher cumulative risk of developing CVD 14 years after the index date (log-rank test, P<0.001).

As indicated in Table 2, at the end of the 14-year follow-up period, patients with AUD had significantly higher incidences of CVD (27.39% vs 19.97%, P<0.001) and several comorbidities than did controls without AUD.

**Table 2:** Characteristics of the patient and control groups at the study endpoint.

AUD	Total		With		Without		P
Variables	n	%	n	%	n	%	
**Total**	37,100		7,420	20.00	29,680	80.00	
**CVD**							<0.001
Without	29,140	78.54	5,388	72.61	23,752	80.03	
With	7,960	21.46	2,032	27.39	5,928	19.97	
**Sex**							0.999
Male	34,445	92.84	6,889	92.84	27,556	92.84	
Female	2,655	7.16	531	7.16	2,124	7.16	
**Age (y)**	49.18 ± 14.48		49.33 ± 12.51		49.14 ± 14.94		0.329
**Age groups (y)**							<0.001
18–24	524	1.41	52	0.70	472	1.59	
25–44	15,783	42.54	2,963	39.93	12,820	43.19	
45–64	14,981	40.38	3,496	47.12	11,485	38.70	
≥65	5,812	15.67	909	12.25	4,903	16.52	
**Low-income**							<0.001
Without	36,092	97.28	7,048	94.99	29,044	97.86	
With	1,008	2.72	372	5.01	636	2.14	
**Catastrophic illness**							<0.001
Without	29,827	80.40	5,417	73.01	24,410	82.24	
With	7,273	19.60	2,003	26.99	5,270	17.76	
**DM**							<0.001
Without	32,560	87.76	6,298	84.88	26,262	88.48	
With	4,540	12.24	1,122	15.12	3,418	11.52	
**HTN**							<0.001
Without	32,226	86.86	6,607	89.04	25,619	86.32	
With	4,874	13.14	813	10.96	4,061	13.68	
**Hyperlipidemia**							<0.001
Without	35,790	96.47	7,209	97.16	28,581	96.30	
With	1,310	3.53	211	2.84	1,099	3.70	
**Obesity**							0.030
Without	37,072	99.92	7,419	99.99	29,653	99.91	
With	28	0.08	1	0.01	27	0.09	
**Liver cirrhosis**							<0.001
Without	32,626	87.94	4,999	67.37	27,627	93.08	
With	4,474	12.06	2,421	32.63	2,053	6.92	
**CKD**							<0.001
Without	35,413	95.45	7,008	94.45	28,405	95.70	
With	1,687	4.55	412	5.55	1,275	4.30	
**COPD**							<0.001
Without	34,693	93.51	6,736	90.78	27,957	94.19	
With	2,407	6.49	684	9.22	1,723	5.81	
**Tobacco use disorder**							0.002
Without	37,089	99.97	7,413	99.91	29,676	99.99	
With	11	0.03	7	0.09	4	0.01	
**Drug use disorder**							<0.001
Without	37,066	99.91	7,404	99.78	29,662	99.94	
With	34	0.09	16	0.22	18	0.06	
**Anxiety**							0.075
Without	36,936	99.56	7,378	99.43	29,558	99.59	
With	164	0.44	42	0.57	122	0.41	
**Depression**							<0.001
Without	36,677	98.86	7,173	96.67	29,504	99.41	
With	423	1.14	247	3.33	176	0.59	
**CCI_R**	0.20 ± 0.52		0.37 ± 0.60		0.16 ± 0.48		<0.001
**Season**							0.124
Spring (Mar–May)	9,046	24.38	1,808	24.37	7,238	24.39	
Summer (Jun–Aug)	9,458	25.49	1,816	24.47	7,642	25.75	
Autumn (Sep–Nov)	9,600	25.88	1,959	26.40	7,641	25.74	
Winter (Dec–Feb)	8,996	24.25	1,837	24.76	7,159	24.12	
**Location**							<0.001
Northern Taiwan	14,573	39.28	2,759	37.18	11,814	39.80	
Middle Taiwan	10,450	28.17	2,180	29.38	8,270	27.86	
Southern Taiwan	9,354	25.21	1,706	22.99	7,648	25.77	
Eastern Taiwan	2,564	6.91	730	9.84	1,834	6.18	
Outlets islands	159	0.43	45	0.61	114	0.38	
**Urbanization level**							<0.001
1 (highest)	12,217	32.93	2,152	29.00	10,065	33.91	
2	15,618	42.10	3,152	42.48	12,466	42.00	
3	3,140	8.46	661	8.91	2,479	8.35	
4 (lowest)	6,125	16.51	1,455	19.61	4,670	15.73	
**Level of care**							<0.001
Hospital center	12,053	32.49	2,046	27.57	10,007	33.72	
Regional hospital	14,788	39.86	3,244	43.72	11,544	38.89	
Local hospital	10,259	27.65	2,130	28.71	8,129	27.39	
**Mortality**							<0.001
Without	34,361	92.62	6,376	85.93	27,985	94.29	
With	2,739	7.38	1,044	14.07	1,695	5.71	

P: Chi-squared/Fisher’s exact test for categorical variables and t-test for continuous variables.

AUD = Alcohol use disorder, CVD = cardiovascular disease, DM = diabetes mellitus, HTN = hypertension, COPD = chronic obstructive pulmonary disease, CKD = chronic kidney disease, CCI = Charlson comorbidity index.

Patients with AUD also exhibited a significantly higher incidence of CVD than did controls without AUD, according to the Cox regression analysis (adjusted HR [AHR] = 1.447, 95% CI = 1.372–1.525, P<0.001). In addition, male sex (AHR = 1.206, 95% CI = 1.096–1.327, P<0.001), DM (AHR = 1.363, 95% CI = 1.293–1.437, P<0.001), HTN (AHR = 1.699, 95% CI = 1.615–1.787, P<0.001), hyperlipidemia (AHR = 1.869, 95% CI = 1.735–2.012, P<0.001), CKD (AHR = 1.395, 95% CI = 1.273–1.529, P<0.001), COPD (AHR = 0.883, 95% CI = 0.810–0.963, P<0.001), anxiety (AHR = 2.044, 95% CI = 1.597–2.616, P<0.001), depression (AHR = 1.642, 95% CI = 1.510–1.807, P<0.001), and CCI (AHR = 1.262, 95% CI = 1.215–1.312, P<0.001) were associated with an increased risk of CVD development (Table 3).

**Table 3:** Risk factors for cardiovascular disease according to Cox regression analysis.

Variables	Crude HR	95% CI	95% CI	P	Adjusted HR	95% CI	95% CI	P
**AUD**								
Without	Reference				Reference			
With	1.334	1.268	1.403	<0.001	1.447	1.372	1.525	<0.001
**Sex**								
Male	1.266	1.151	1.392	<0.001	1.206	1.096	1.327	<0.001
Female	Reference				Reference			
**Age groups (y)**								
18–24	Reference				Reference			
25–44	1.106	0.626	1.952	0.728	1.032	0.528	1.647	0.809
45–64	1.478	0.838	2.605	0.177	1.095	0.621	1.933	0.753
≥65	2.160	1.225	3.811	0.008	1.511	0.856	2.668	0.155
**Low-income**								
Without	Reference				Reference			
With	0.942	0.840	1.056	0.304	1.001	0.891	1.124	0.986
**Catastrophic illness**								
Without	Reference				Reference			
With	1.018	0.967	1.073	0.487	1.001	0.949	1.056	0.963
**DM**								
Without	Reference				Reference			
With	1.730	1.645	1.819	<0.001	1.363	1.293	1.437	<0.001
**HTN**								
Without	Reference				Reference			
With	1.997	1.906	2.092	<0.001	1.699	1.615	1.787	<0.001
**Hyperlipidemia**								
Without	Reference				Reference			
With	2.282	2.124	2.451	<0.001	1.869	1.735	2.012	<0.001
**Obesity**								
Without	Reference				Reference			
With	1.103	0.500	1.631	0.735	1.136	0.407	1.333	0.312
**Liver cirrhosis**								
Without	Reference				Reference			
With	1.111	1.035	1.192	0.004	1.006	0.854	1.204	0.061
**CKD**								
Without	Reference				Reference			
With	1.423	1.300	1.558	<0.001	1.395	1.273	1.529	<0.001
**COPD**								
Without	Reference				Reference			
With	0.943	0.866	1.027	0.178	0.883	0.810	0.963	0.005
**Tobacco use disorder**								
Without	Reference				Reference			
With	1.005	0.021	1.045	0.055	1.025	0.032	1.597	0.136
**Drug use disorder**								
Without	Reference				Reference			
With	1.007	0.109	1.047	0.060	1.064	0.149	1.445	0.185
**Anxiety**								
Without	Reference				Reference			
With	1.771	1.385	2.265	<0.001	2.044	1.597	2.616	<0.001
**Depression**								
Without	Reference				Reference			
With	1.672	1.332	2.099	<0.001	1.642	1.510	1.807	<0.001
**CCI_R**	1.221	1.181	1.262	<0.001	1.262	1.215	1.312	<0.001
**Season**								
Spring (Mar–May)	Reference				Reference			
Summer (Jun–Aug)	0.907	0.852	0.966	0.002	0.929	0.873	0.989	0.021
Autumn (Sep–Nov)	0.814	0.765	0.866	<0.001	0.799	0.751	0.850	<0.001
Winter (Dec–Feb)	0.976	0.917	1.038	0.438	0.944	0.887	1.004	0.069
**Location**					**Multicollinearity with urbanization level**			
Northern Taiwan	Reference				**Multicollinearity with urbanization level**			
Middle Taiwan	1.140	1.079	1.204	<0.001	**Multicollinearity with urbanization level**			
Southern Taiwan	1.121	1.059	1.186	<0.001	**Multicollinearity with urbanization level**			
Eastern Taiwan	1.157	1.063	1.260	0.001	**Multicollinearity with urbanization level**			
Outlets islands	1.003	0.704	1.429	0.987	**Multicollinearity with urbanization level**			
**Urbanization level**								
1 (highest)	0.894	0.838	0.954	0.001	1.042	0.969	1.121	0.266
2	0.995	0.937	1.056	0.861	1.113	1.045	1.186	0.001
3	0.771	0.700	0.848	<0.001	0.800	0.726	0.881	<0.001
4 (lowest)	Reference				Reference			
**Level of care**								
Hospital center	1.938	1.890	1.988	<0.001	1.647	1.605	1.692	<0.001
Regional hospital	1.331	1.256	1.410	<0.001	1.628	1.594	1.664	<0.001
Local hospital	Reference				Reference			

Adjusted HR: Adjusted variables as listed in the table.

HR = hazard ratio, CI = confidence interval, AUD = alcohol use disorder, CVD = cardiovascular disease, DM = diabetes mellitus, HTN = hypertension, COPD = chronic obstructive pulmonary disease, CKD = chronic kidney disease, CCI = Charlson comorbidity index.

Table 4 presents the results of analyses, stratified by demographic factors and comorbidities and Fine and Gray’s competing risk model. The incidence of CVD was higher in the case cohort than in the control cohort (3,801.64 vs. 2,884.75 per 105 person-years), and the overall incidence of CVD was 1.447-fold higher in the case cohort than in the control cohort. The risk of CVD is higher for low-income AUD patients than for those without low-income, compared with those without low-income households (AHR = 3.383; 95% CI = 3.209–3.566; with competing risk model AHR = 2.293, 95% CI = 2.034–2.564, P<0.001). In addition, the risk of CVD was 2.806 times higher in obese patients with AUD, and 2.089 times higher by the competing risk model (AHR = 2.806; 95% CI = 2.662–2.958; With competing risk model AHR = 2.089, 95% CI = 1.853–2.336, P<0.001).

**Table 4:** Risk factors for cardiovascular disease stratified by variables according to Cox regression analysis with/without Fine and Gray’s competing risk model.

AUD	With			Without *(Reference)*			Without competing risk model				With competing risk model			
Stratified	Events	PYs	Rate (per 10^5^ PYs)	Events	PYs	Rate (per 10^5^ PYs)	Adjusted HR	95% CI	95% CI	*P*	Adjusted HR	95% CI	95% CI	*P*
**Total**	2,032	53,450.61	3,801.64	5,928	205,494.31	2,884.75	1.447	1.372	1.525	< 0.001	1.500	1.330	1.677	< 0.001
**Sex**														
Male	1,900	49,093.23	3,870.19	5,599	191,461.56	2,924.35	1.453	1.378	1.532	< 0.001	1.503	1.333	1.681	< 0.001
Female	132	4,357.38	3,029.34	329	14,032.75	2,344.52	1.419	1.346	1.496	< 0.001	1.485	1.317	1.661	< 0.001
**Age groups (y)**														
18–24	3	55.70	5,385.93	28	524.15	5,342.01	1.107	1.050	1.167	0.002	1.312	1.164	1.467	< 0.001
25–44	497	15,474.61	3,211.71	1,245	51,964.65	2,395.86	1.472	1.396	1.552	< 0.001	1.513	1.342	1.691	< 0.001
45–64	1,004	29,798.39	3,369.31	2,513	102,384.35	2,454.48	1.507	1.430	1.589	< 0.001	1.531	1.358	1.712	< 0.001
≥65	528	8,121.90	6,500.94	2,142	50,621.16	4,231.43	1.687	1.600	1.778	< 0.001	1.619	1.437	1.811	< 0.001
**Low-income**														
Without	1,841	49,929.71	3,687.18	5,813	198,962.74	2,921.65	1.386	1.314	1.461	< 0.001	1.468	1.302	1.641	< 0.001
With	191	3,520.90	5,424.75	115	6,531.57	1,760.68	3.383	3.209	3.566	< 0.001	2.293	2.034	2.564	< 0.001
**Catastrophic illness**														
Without	1,265	37,050.17	3,414.29	4,510	161,935.27	2,785.06	1.346	1.277	1.419	< 0.001	1.446	1.283	1.618	< 0.001
With	767	16,400.43	4,676.71	1,418	43,559.05	3,255.35	1.577	1.496	1.663	< 0.001	1.566	1.389	1.751	< 0.001
**DM**														
Without	1,397	43,942.70	3,179.14	4,309	171,996.76	2,505.28	1.393	1.322	1.469	< 0.001	1.472	1.306	1.646	< 0.001
With	635	9,507.91	6,678.65	1,619	33,497.56	4,833.19	1.517	1.439	1.599	< 0.001	1.536	1.362	1.717	< 0.001
**HTN**														
Without	1,448	45,320.96	3,194.99	3,697	161,055.46	2,295.48	1.528	1.450	1.611	< 0.001	1.541	1.367	1.724	< 0.001
With	584	8,129.65	7,183.58	2,231	44,438.85	5,020.38	1.571	1.490	1.656	< 0.001	1.563	1.386	1.748	< 0.001
**Hyperlipidemia**														
Without	1,818	51,537.74	3,527.51	5,199	194,566.19	2,672.10	1.449	1.375	1.528	< 0.001	1.501	1.332	1.679	< 0.001
With	214	1,912.87	11,187.39	729	10,928.13	6,670.86	1.841	1.747	1.941	< 0.001	1.692	1.501	1.892	< 0.001
**Obesity**														
Without	2,031	53,436.82	3,800.75	5,918	205,141.82	2,884.83	1.447	1.372	1.525	< 0.001	1.500	1.330	1.677	< 0.001
With	1	13.79	7,251.07	10	352.50	2,836.92	2.806	2.662	2.958	< 0.001	2.089	1.853	2.336	< 0.001
**Liver cirrhosis**														
Without	1,506	40,431.30	3,724.84	5,594	192,819.39	2,901.16	1.410	1.337	1.486	< 0.001	1.480	1.313	1.655	< 0.001
With	526	13,019.31	4,040.15	334	12,674.92	2,635.12	1.683	1.597	1.775	< 0.001	1.618	1.435	1.809	< 0.001
**CKD**														
Without	1,865	50,571.41	3,687.85	5,530	196,456.66	2,814.87	1.438	1.364	1.516	< 0.001	1.495	1.327	1.672	< 0.001
With	167	2,879.20	5,800.22	398	9,037.65	4,403.80	1.446	1.372	1.525	< 0.001	1.499	1.330	1.677	< 0.001
**COPD**														
Without	1,882	48,125.71	3,910.59	5,510	191,156.62	2,882.45	1.490	1.413	1.570	< 0.001	1.522	1.350	1.702	< 0.001
With	150	5,324.89	2,816.96	418	14,337.69	2,915.39	1.061	1.006	1.118	0.043	1.284	1.139	1.436	< 0.001
**Tobacco use disorder**														
Without	2,031	53,322.17	3,808.92	5,928	205,430.44	2,885.65	1.449	1.375	1.528	< 0.001	1.501	1.332	1.678	< 0.001
With	1	128.44	778.57	0	63.88	0.00	∞	-	-	0.898	∞	-	-	0.886
**Drug use disorder**														
Without	2,030	53,332.33	3,806.32	5,926	205,337.62	2,885.98	1.448	1.374	1.527	< 0.001	1.500	1.331	1.678	< 0.001
With	2	118.28	1,690.90	2	156.69	1,276.38	1.454	1.380	1.533	< 0.001	1.504	1.334	1.681	< 0.001
**Anxiety**														
Without	2,009	53,153.43	3,779.62	5,881	204,592.76	2,874.49	1.444	1.369	1.522	< 0.001	1.498	1.329	1.675	< 0.001
With	23	297.18	7,739.52	47	901.56	5,213.21	1.630	1.546	1.718	< 0.001	1.592	1.412	1.780	< 0.001
**Depression**														
Without	1,963	51,368.56	3,821.40	5,888	203,648.32	2,891.26	1.451	1.376	1.530	< 0.001	1.502	1.332	1.680	< 0.001
With	69	2,082.05	3,314.04	40	1,845.99	2,166.86	1.679	1.593	1.770	< 0.001	1.616	1.433	1.807	< 0.001
**Season**														
Spring (Mar–May)	563	12,838.73	4,385.17	1,467	48,180.82	3,044.78	1.581	1.500	1.667	< 0.001	1.568	1.391	1.753	< 0.001
Summer (Jun–Aug)	441	12,373.98	3,563.93	1,471	52,006.30	2,828.50	1.383	1.312	1.458	< 0.001	1.466	1.301	1.640	< 0.001
Autumn (Sep–Nov)	503	15,196.56	3,309.96	1,510	56,803.93	2,658.27	1.367	1.297	1.441	< 0.001	1.458	1.293	1.630	< 0.001
Winter (Dec–Feb)	525	13,041.33	4,025.66	1,480	48,503.26	3,051.34	1.448	1.374	1.527	< 0.001	1.501	1.331	1.678	< 0.001
**Urbanization level**														
1 (highest)	529	14,666.80	3,606.79	1,741	63,300.22	2,750.39	1.440	1.366	1.518	< 0.001	1.496	1.327	1.673	< 0.001
2	976	22,981.01	4,246.98	2,704	88,191.25	3,066.06	1.521	1.443	1.603	< 0.001	1.538	1.364	1.719	< 0.001
3	162	5,037.67	3,215.77	485	17,574.24	2,759.72	1.279	1.214	1.349	< 0.001	1.410	1.251	1.577	< 0.001
4 (lowest)	365	10,765.12	3,390.58	998	36,428.61	2,739.60	1.359	1.289	1.433	< 0.001	1.453	1.289	1.625	< 0.001
**Level of care**														
Hospital center	633	14,984.41	4,224.39	1,952	67,396.82	2,896.28	1.601	1.519	1.688	< 0.001	1.578	1.400	1.764	< 0.001
Regional hospital	854	25,476.91	3,352.05	2,488	95,137.59	2,615.16	1.407	1.335	1.484	< 0.001	1.479	1.312	1.654	< 0.001
Local hospital	545	12,989.29	4,195.77	1,488	42,959.90	3,463.70	1.330	1.262	1.402	< 0.001	1.438	1.276	1.608	< 0.001

Adjusted HR: The multivariable analysis included sex, age, covariates, and comorbidities (hypertension [HTN], diabetes mellitus [DM], hyperlipidemia, ischemic heart diseases, congestive heart failure, chronic obstructive pulmonary disease [COPD], liver disease, rheumatic disease, connective tissue disease, multiple sclerosis, osteoporosis).

PYs = person-years, adjusted HR = adjusted hazard ratio (adjusted for the variables listed in Table 3), CI = confidence interval, AUD = alcohol use disorder, CVD = cardiovascular disease, CKD = chronic kidney disease.

We categorized the CVD cohort into CVD subgroups according to the ICD-9-CM codes. Table 5 shows that patients with AUD in different CVD subgroups, such as CVD, IHD, and stroke, were at a significantly higher risk than those without AUD: CVD (AHR = 1.447, 95% CI = 1.372–1.525, P<0.001), IHD (AHR = 1.304, 95% CI = 1.214–1.401, P<0.001), and stroke (AHR = 1.640, 95% CI = 1.519–1.770, P<0.001). Moreover, our findings revealed significant differences in the risks of CVD, IHD, and stroke among subgroups with and without AUD. Of note, in the AUD-stratified analysis, the effects of alcohol abuse on the risk of CVD, IHD, and stroke were not significantly different, similar to the results of the competing risk model: CVD (AHR = 1.500, 95% CI = 1.330–1.677, P<0.001), IHD (AHR = 1.424, 95% CI = 1.251–1.607, P<0.001), and stroke (AHR = 1.596, 95% CI = 1.400–1.806, P<0.001). These results show the importance of abstinence from alcohol.

**Table 5:** Sensitivity for factors of CVD subgroups among different AUD types by Cox regression analysis with/without Fine and Gray’s competing risk model.

							Without competing risk model				With competing risk model			
Sensitivity test	CVD subgroups	AUD types	Population	Events	PYs	Rate (per 10^5^ PYs)	Adjusted HR	95% CI	95% CI	*P*	Adjusted HR	95% CI	95% CI	*P*
**Any of the listed**	CVD	Without AUD	29,680	5,928	205,494.31	2,884.75	Reference							
		With AUD	7,420	2,032	53,450.61	3,801.64	1.447	1.372	1.525	< 0.001	1.500	1.330	1.677	< 0.001
		Alcoholic psychoses	2,313	621	15,806.69	3,928.72	1.515	1.391	1.650	< 0.001	1.535	1.339	1.744	< 0.001
		Alcohol dependence	4,862	1,358	35,631.90	3,811.19	1.434	1.350	1.524	< 0.001	1.493	1.319	1.676	< 0.001
		Alcohol abuse	245	53	2,012.02	2,634.17	1.139	0.869	1.494	0.347	1.331	0.958	1.660	0.163
	IHD	Without AUD	29,680	3,531	205,494.31	1,718.30	Reference							
		With AUD	7,420	1,084	53,450.61	2,028.04	1.304	1.214	1.401	< 0.001	1.424	1.251	1.607	< 0.001
		Alcoholic psychoses	2,313	300	15,806.69	1,897.93	1.282	1.135	1.448	< 0.001	1.412	1.210	1.634	< 0.001
		Alcohol dependence	4,862	757	35,631.90	2,124.50	1.331	1.228	1.444	< 0.001	1.439	1.258	1.632	< 0.001
		Alcohol abuse	245	27	2,012.02	1,341.94	0.938	0.642	1.371	0.742	1.208	0.810	1.590	0.484
	Stroke	Without AUD	29,680	2,557	205,494.31	1,244.32	Reference							
		With AUD	7,420	993	53,450.61	1,857.79	1.640	1.519	1.770	< 0.001	1.596	1.400	1.806	< 0.001
		Alcoholic psychoses	2,313	339	15,806.69	2,144.66	1.829	1.627	2.056	< 0.001	1.686	1.449	1.947	< 0.001
		Alcohol dependence	4,862	626	35,631.90	1,756.85	1.565	1.431	1.712	< 0.001	1.560	1.359	1.777	< 0.001
		Alcohol abuse	245	28	2,012.02	1,391.64	1.439	0.990	2.091	0.056	1.496	1.003	1.964	0.048
**Respectively**	IHD	Without AUD	29,680	4,016	207,458.20	1,935.81	Reference							
		With AUD	7,420	1,253	54,091.79	2,316.43	1.330	1.245	1.422	< 0.001	1.438	1.267	1.619	< 0.001
		Alcoholic psychoses	2,313	354	15,993.44	2,213.41	1.330	1.189	1.487	< 0.001	1.438	1.238	1.656	< 0.001
		Alcohol dependence	4,862	865	36,070.83	2,398.06	1.345	1.247	1.451	< 0.001	1.446	1.268	1.636	< 0.001
		Alcohol abuse	245	34	2,027.52	1,676.93	1.039	0.741	1.458	0.823	1.271	0.878	1.640	0.359
	Stroke	Without AUD	29,680	3,076	207,938.71	1,479.28	Reference							
		With AUD	7,420	1,178	54,275.30	2,170.42	1.645	1.534	1.765	< 0.001	1.599	1.407	1.804	< 0.001
		Alcoholic psychoses	2,313	385	16,001.33	2,406.05	1.794	1.609	2.001	< 0.001	1.670	1.440	1.921	< 0.001
		Alcohol dependence	4,862	765	36,261.95	2,109.65	1.602	1.477	1.737	< 0.001	1.578	1.380	1.790	< 0.001
		Alcohol abuse	245	28	2,012.02	1,391.64	1.219	0.839	1.770	0.299	1.376	0.940	1.806	0.191

PYs = person-years, adjusted HR = adjusted hazard ratio (adjusted for the variables listed in Table 3), CI = confidence interval, IHD = ischemic heart disease, CVD = cardiovascular disease, AUD = alcohol use disorder.

## Discussion

Alcohol has a strong effect on the human body and mind, even at low doses; its neurotoxic, hepatotoxic, and carcinogenic properties make it a potent risk factor for disease burden [7]. To the best of our knowledge, this is the first national cohort study to establish a substantial correlation between AUD and CVD. The results indicate that patients with AUD have an increased risk of CVD. In addition, the risk of developing IHD and stroke was significantly higher in patients with AUD than in those without AUD.

Several epidemiological studies published in the previous three decades have reported a cardio protective effect of low-to-moderate alcohol intake; however, the number of published studies alone is not an indicator of the strength of the evidence on this effect, let alone a causal effect. Many drinkers cite health benefits, mainly for cardio-protection, as a reason for drinking alcohol, despite often-raised concerns in the scientific literature regarding the causality of a cardio protective effect.

The effect of alcohol on the risk of IHD also makes this an intriguing and sometimes controversial topic in terms of disease epidemiology and public policy. The quality of epidemiological studies has substantially improved in the previous three decades. However, several studies have used recent abstainers as the reference group, and this can lead to systematic bias and erroneous conclusions; hence, high-quality epidemiological evidence is needed to provide a clear picture of the topic. When examining average alcohol consumption in comparison to lifetime abstinence, the relationship between alcohol consumption and IHD risk follows a J-curve. The curve shows a more detrimental association with lower average alcohol levels for women than for men [8]. This is consistent with our literature, which indicates that patients with AUD have a higher mortality rate than those without AUD, and the gender component is also consistent with the literature.

Average alcohol consumption alone is not sufficient to describe the alcohol-IHD relationship. Drinking patterns play an important role, and both episodic and chronic heavy drinking may negate any beneficial effect of alcohol consumption on IHD risk and even elevate the risk substantially. Nevertheless, in several epidemiological and short-term experimental studies, relative to lifetime abstinence, having one to two drinks per drinking day without episodic heavy drinking showed a beneficial association with the risk of IHD [8]. The alcohol-IHD relationship fulfills the criteria for a causal association as proposed by Hill [9]. Whether one detects an inverse, U-shaped, or J-shaped relationship depends on the distribution of drinking patterns in a given population. The prevalence of heavy drinking patterns has been on the rise in several countries, such as Canada, the US, the UK, and several Eastern European and Asian countries [10–13]. In the US, episodic heavy drinking is more common than chronic heavy drinking [13]. In our study, we showed that alcoholic psychoses, alcohol dependence and CVD (IHD, stroke) were significantly related, but alcohol abuse was not significantly related to CVD (IHD, stroke), which we believe is because alcohol abuse is likely to cause death before CVD is diagnosed.

Furthermore, the overall risk-benefit relationship of any form of alcohol consumption on an individual level must be judged cautiously in light of the well-known detrimental effects of alcohol use on other disease outcomes, such as injuries and cancer [3,14,15]. Hence, making recommendations for clinical practice is challenging because of the simultaneous beneficial and detrimental effects of, on average, low alcohol consumption, and because evidence from randomized controlled trials on the long-term effects of alcohol consumption is lacking. This has been confirmed in our study that chronic diseases are positively correlated in patients with AUD.

There is no control mechanism for alcohol purchase as there is for prescription drugs because alcohol is freely available for self- and over-medication. Therefore, alcohol consumption should not be considered an option for the prevention of IHD. In terms of public alcohol policy, the picture is clear: alcohol consumption should be as low as possible, no amount of consumption is safe, and episodic and chronic heavy drinking should be strongly discouraged [16,17].

There are two major stroke subtypes with differing etiologies: ischemic stroke (IS) (based on ischemic disease processes) and hemorrhagic stroke (HS) (based on hemorrhagic processes, i.e., bleeding processes). Given the higher prevalence of IS than HS, IS typically drives investigations on stroke. With similarities in etiologies, one would expect IS to show a similar relationship with alcohol consumption as IHD. Indeed, several studies have demonstrated that the association between average alcohol consumption and IS follows a J-curve [18–21]. The risk for intracerebral and subarachnoid HS increased with every drink, and the consumption of >48 g per day resulted in a relative risk of 1.67 (95% CI: 1.25–2.23) for intracerebral stroke and 1.82 (95% CI: 1.18–2.82) for subarachnoid HS [20,22]. One study suggested that heavy alcohol intake is associated with an increased risk of stroke and that low-to-moderate alcohol intake may be protective against total and IS risk [23]. Another study suggested that an alcohol intake of <15 g/day is associated with a reduced risk of total stroke and stroke mortality [24]. This point echoes our study.

Epidemiological studies indicate a complex relationship between various dimensions of alcohol consumption and CVD outcomes. Most epidemiological studies have relied on a single measurement of alcohol intake at baseline. It is assumed that the self-reported drinking levels, including drinking patterns, preferably remain the same before and after the baseline measurement; however, this is not the case for many people, and even lifetime abstainers are difficult to identify [25]. However, in our study, we found that alcoholic psychoses and alcohol dependence were significantly associated with CVD, and alcohol abuse was associated with a high mortality rate; hence, our results indicate the importance of initial abstinence from alcohol.

This study has some limitations. Although the study extensively adjusted the multivariate logistic regression models, there may still be residual confounders. First, the NHIRD does not provide detailed information on variables such as socioeconomic factors, occupation, unhealthy behaviors, amount of alcohol consumption, and the genetic background of the subjects. In addition, the NHIRD does not collect data on sleep quantity. A previous study found that sleep duration may be a risk factor for future alcohol-related diseases [26]. Additionally, the study participants were selected on the basis of their medical records in the NHIRD. When patients with CVDs or AUD choose not to undergo treatment in the hospital, their data are not recorded in the NHIRD; hence, many cases may be missed. Finally, AUD may be divided into different stages based on the patient’s temporal exposure to alcohol; this study did not take the stage of alcohol use into account. Thus, our results may have underestimated the prevalence of CVDs and AUD.

## Conclusion

This study found a significantly higher risk of diagnosis for CVD in patients with AUD, and we also observed an association between alcohol-related diseases and the development of CVD even after adjusting for several comorbidities and sensitivity test in a nationwide cohort. If the association reflects a causal effect, these findings strongly suggest that clinicians should inform the patients about the risk of CVD and the benefits of quitting alcohol and that the earlier you stop drinking, the better the cardiovascular benefits.

## Supporting information

S1 Table Abbreviation and ICD-9-CM. (DOCX)
