# Supplementary material for: Risk of cardiovascular disease in patients with alcohol use disorder: A population-based retrospective cohort study
Source: PLoS One. 2022 Oct 25;17(10):e0276690. doi: 10.1371/journal.pone.0276690 (PMC9595521; doi:10.1371/journal.pone.0276690)
Supplement: S1 Table — (DOCX) [file pone.0276690.s001.docx]

**Risk of cardiovascular disease in patients with alcohol use disorder: A population-based retrospective cohort study**

**SUPPORTING INFORMATION**

| **Table S1. Abbreviation and ICD-9-CM** | | |
| --- | --- | --- |
|  | **Abbreviation** | **ICD-9-CM / Definition** |
| **Study population:** Alcohol use disorders | AUD | Outpatient visits ≥3 or inpatient; Priority: Alcohol abuse, Alcohol dependence, and Alcoholic psychoses |
| Alcoholic psychoses |  | 291 |
| Alcohol dependence |  | 303 |
| Alcohol abuse |  | 305.0 |
| **Events:** Cardiovascular disease | CVD |  |
| Ischemic heart disease | IHD | 410–414 |
| Stroke |  | 430–438 |
| **Comorbidities** |  |  |
| Diabetes mellitus | DM | 250 |
| Hypertension | HTN | 401–405 |
| Hyperlipidemia |  | 272 |
| Obesity |  | 278 |
| Liver cirrhosis |  | 571 |
| Chronic kidney disease | CKD | 580–589 |
| Chronic obstructive pulmonary disease | COPD | 480–486 |
| Tobacco use disorder |  | 350.1 |
| Drug use disorder |  | 304, 305.2–305.9 |
| Anxiety |  | 300.0 |
| Depression |  | 296.2–296.3, 300.4, 311 |
| **Charlson Comorbidity Index Revised** | CCI_R | CCI removed CVD, DM, HTN, Liver cirrhosis, CKD, and COPD |
